# Upper airway changes after rapid maxillary expansion: three-dimensional analyses

**DOI:** 10.1186/s12903-023-03324-0

**Published:** 2023-08-31

**Authors:** Carlos de Julián-López, Jesús Veres, Laura Marqués-Martínez, Esther García-Miralles, Santiago Arias, Clara Guinot-Barona

**Affiliations:** 1 Orthodontics, Dentistry Department, Faculty of Medicine and Health Sciences, University CEU-Cardenal Herrera, C/Del Pozo s/n, Alfara del Patriarca, Valencia, 46115 Spain; 2 Dentistry Department, Faculty of Medicine and Health Sciences, Catholic University of Valencia San Vicente Mártir, Valencia, 46001 Spain

**Keywords:** Upper airway, Rapid maxillary expansion, Sleep apnea disorder

## Abstract

The objective of this study was to evaluate volumetric changes in the upper airway using Cone Beam Computed Tomography (CBCT) in orthodontic patients with maxillary transversal hypoplasia undergoing maxillary disjunction. The influence of factors such as sex, age, and growth pattern on airway volumetric changes was also assessed. The sample consisted of 50 growing patients from the dental clinic of Cardenal Herrera CEU University of Valencia. Airway volume was measured in mm3 before treatment (T0) and after palatal disjunction (T1). The final sample included 37 subjects in the treatment group and 13 in the control group. The volume gained exclusively from the disjunction treatment was determined to differentiate it from natural growth. The control group showed a mean volume increase from 10,911.3 ± 1,249.6 mm3 to 13,168.9 ± 1,789.7 mm3, representing a mean increase of 2,257.6 mm3 or + 20.9%. The treatment group exhibited an increase from 14,126.3 ± 4,399.8 mm3 at T0 to 18,064.1 ± 4,565.9 mm3 at T1, corresponding to a gain of 3,937.8 mm3 or + 31.8%. Significant differences in airway volume were observed after palatal disjunction compared to the control group. The expansion of the maxilla led to a significant increase in airway volume in the treated patients, estimated at 5,183 mm3 (+ 41.5%).

## Introduction

Pediatric obstructive sleep apnea is characterized by respiratory rhythm disorders during sleep. It is primarily caused by upper airway resistance or obstruction, leading to inadequate ventilation and pulmonary oxygenation, resulting in poor sleep quality [1–4]. In children, the primary cause of respiratory problems is often the obstruction of the upper airway by lymphatic tissue (adenoid-tonsillar hypertrophy), which undergoes rapid growth in the first three years of life, followed by a slower growth rate and involution after puberty [1, 4–6].

Numerous studies have explored the relationship between naso respiratory function, craniofacial morphology, and the etiology of apnea and malocclusion in children. Respiratory pathology in the upper airway, combined with oral breathing, can contribute to specific malocclusions and facial morphologies. Adenoid pathology influences the development of oral breathing in individuals with certain facial characteristics and malocclusions [5–8].

Airway obstruction affects craniofacial development, although the extent of these changes varies among individuals and does not determine specific dentofacial alterations. Chronic oral breathing plays a significant role in craniofacial structure development, resulting in malocclusions and cranial abnormalities such as increased facial height, maxillary atresia, maxillary underdevelopment, or posterior crossbite, particularly deficient transverse development of the maxilla. Early orthodontic intervention is crucial for addressing these common occlusal abnormalities [4, 7].

Rapid maxillary expansion (RME) is a commonly used orthodontic technique for correcting transverse deficiencies of the maxilla resulting from underdevelopment during the patient’s growth period. As the hard palate is closely associated with the nasal cavity, maxillary expansion also leads to an expansion of the nasal upper airway [6, 9]. RME widens the palate, flattens the palatal arch with inferior displacement of the maxilla, and influences mandibular alignment [9]. Furthermore, RME has been found to have a positive impact on respiratory function and can contribute to the reduction of respiratory diseases. By widening the nasal airway and reducing air resistance, RME helps restore natural physiological function and improve overall respiratory health [6, 10–14].

This statement is supported by the observation that the maxillary bones comprise approximately 50% of the structures within the nasal cavity and the upper airway, while the nasal cavity alone contributes to half of the overall resistance in the upper airway [11, 15].

Cone Beam Computed Tomography (CBCT) currently stands as the most commonly employed technology for acquiring digital data regarding the specific anatomy of the nose and pharynx in dentistry [15, 16]. The utilization of software for three-dimensional reconstruction and image manipulation across three spatial planes has facilitated research focusing on the volume and morphology of the upper airway in relation to its growth. Consequently, investigations into the dentoskeletal effects of rapid maxillary expansion have been extensively conducted, utilizing various techniques ranging from manual cast measurement to lateral skull radiographs [16, 17]. The integration of CBCT into studies has significantly enhanced the ability to obtain comprehensive information about the airway and nasal anatomy. As a result, there is an increasing interest in conducting these tests, owing to the expanding importance of sleep medicine and its association with rapid maxillary expansion [16, 17].

Based on this, the objective of the research is to assess the short-term volumetric changes that occur following rapid maxillary expansion treatment in growing patients.

## Materials and methods

The study was conducted in accordance with the Declaration of Helsinki and approved by Universidad CEU Cardenal Herrera Ethics Committee (protocol code CEI18/185 approved on 20 December 2018). Informed consent was obtained from all subjects and their parents/legal guardian involved in the study.

### Study design

After obtaining approval from the board at the Cardenal Herrera CEU University of Valencia (CEI18/185), a retrospective case-control study was conducted. Consecutive cases that met the inclusion criteria were selected, and their diagnoses were aided by CBCT imaging. The study included growing patients diagnosed with maxillary hypoplasia who underwent airway volume assessment using CBCT and were treated with a Hyrax maxillary expander. Patients with congenital malformations, those undergoing surgical disjunction treatment, and those treated with a disjunction protocol and facemask or functional appliances were excluded.

All patient records meeting the inclusion criteria and who visited the dental clinic at the Cardenal Herrera CEU University of Valencia for an orthodontic study were analyzed. Based on CBCT assessment using a mathematical method, it was determined that these patients had a diagnosis of skeletal compression of the maxilla [18]. The sample underwent treatment with rapid maxillary expansion using a tooth-supported device. A second CBCT was performed within 24 months after the device was blocked to evaluate changes.

The final sample consisted of 50 patients, and their airway volumes were measured in mm3 at two time points: before treatment (T0) and after palatal disjunction and retention (T1). Out of the total sample, 37 patients were in the treatment group, and 13 were in the control group. Among the total sample, 24 patients were male, and 26 were female. Within the treatment group, 19 were male and 18 were female, while in the control group, 5 were male and 8 were female.

CBCT scans were performed with the patient seated and their head in the natural head position. Patients were instructed to occlude their teeth in maximum intercuspation, relax their tongues, and breathe calmly. The effective absorption dose was 57 microsieverts, and the scanning time was 36 s. This corresponds to a radiation dose of 6 microsieverts, which is comparable to a periapical series of all teeth and can range from 33 to 84 microsieverts depending on speed, technique, and kilovoltage.

The sample was divided into three chronological age groups: under 10 years, between 10 and 13 years, and over 13 years (Table 1).

**Table 1:** Sample groups attending the chronological age

			GROUP			
	TOTAL		CONTROL		TREATMENT	
	N	%	N	%	N	%
TOTAL	50	110%	13	100%	37	100%
< 10 years old	20	40.0%	5	38.5%	15	40.5%
10–13 years old	19	38.0%	4	30.8%	15	40.5%
> 13 years old	11	22.0%	4	30.8%	7	18.9%

**Fig. 1:**
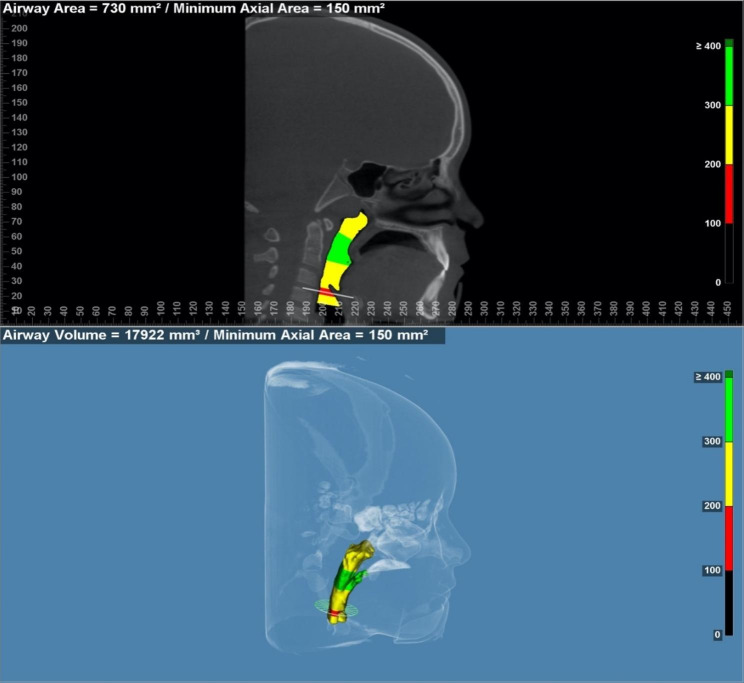
Reproduction of the airway using the Dolphin software

After acquiring the CBCT T0 scans, the DICOM (Digital Imaging and Communications in Medicine) data was imported into the Dolphin Imaging® software for further analysis. Using the software, a 3D reconstruction of the airways was generated (Fig. 1). Subsequently, the volume of the airways was calculated using the software’s measurement tools.

In terms of data management, the orientation of the skull was adjusted to be parallel to the Frankfort plane, following the method described by Guijarro-Martínez in 2013 [17, 19]. To establish the reference planes, specific landmarks were used.

The midsagittal plane was determined by fixing it through the center of the anterior nasal spine. The axial plane was constructed by passing through both infraorbital points. For the right sagittal reference, the axial plane was positioned through the porion and the right infraorbital point. The left sagittal reference was not used to prevent orientation issues caused by the asymmetry of the points.

Regarding the transverse orientation, the median sagittal plane was created by connecting the cresta galli (located at the top of the nasal cavity) and the basion point (a landmark on the anterior cranial base). This allowed for consistent and standardized orientation of the skull for further analysis and measurements.

After orienting the skull, the volume of the upper airway was assessed by identifying and marking the anatomical boundaries of the three regions: nasopharynx, oropharynx, and hypopharynx. These boundaries were defined within the software program.

By establishing the anatomical limits of the pharynx in the software, the volume of the upper airway was automatically calculated and expressed in mm3 (cubic millimeters) [17]. This measurement allowed for quantitative assessment of the changes in airway volume before and after the rapid maxillary expansion treatment.

### Statistical analysis

The statistical analysis of the data involved both descriptive statistics and comparisons of the absolute values of airway volumes between the two groups, taking into account profile factors such as sex, age, and facial pattern.

To assess the normality of airway measurements at T0 and T1 within the groups, the Shapiro-Wilk test was used, and it yielded a confirmatory result (p < 0.05). This indicates that a parametric approach can be used to address the research objectives.

The multivariate analysis involved fitting a General Linear Model of Repeated Measures for the response variable “Airway Volume.“ This model included a within-subjects factor (time: T0, T1) and a between-subjects factor (group: test/control). Bonferroni multiple comparison tests were conducted to evaluate differences between groups at each time point and between time points within each group.

Furthermore, a multiple linear regression model was estimated to explain the final volume at T1 for the control group. The model included baseline volume, follow-up time, sex, age, and facial pattern as predictor variables. The paired t-test was used to compare the means of the final volume with the volume predicted by the regression equation in the treated group. The non-parametric Mann-Whitney and Kruskal-Wallis tests were used to assess whether any additional gain in volume due to treatment was homogeneous across sex, age group, or facial pattern.

A significance level of 5% (α = 0.05) was used in all analyses. The achieved power for testing within-subject effects in the ANOVA model was 0.98, and for between-subject effects, it was 0.80. This indicates a high power to detect statistically significant differences if the pattern of change in airway volumes between the two groups is clearly different.

Overall, the statistical analysis employed various tests and models to comprehensively evaluate the differences and patterns of change in airway volumes between the test and control groups, considering various factors such as time, group, and individual characteristics.

### Method error

To assess the reliability and reproducibility of the measurement technique, the method error was calculated by comparing the intra-examiner measurement differences between the first and second measurements. The reliability was quantified using the CCI (Concordance Correlation Coefficient), which provides an indication of the reproducibility of the measurements.

In Table 2, the CCI values obtained were high, exceeding 0.90. This indicates a high level of concordance and reproducibility of the measurement technique. The high CCI values confirm that the main investigator, who performed the measurements, had consistent and reliable results, with minimal measurement error.

Based on these findings, it can be concluded that the measurement technique used in the study demonstrated a high level of reproducibility, suggesting that the measurements were reliable and consistent throughout the study.

**Table 2:** The intra-observer reproducibility of the measurement method was assessed, and the basic statistics for the differences between the measurements at T0 and T1, as well as those related to the method error, are presented below

	Media	SD	D	CV (%)	CCI
Volume T0	28,2	477,8	332,6	2,64	0,99
Volume T1	236,0	713,4	523,0	3,19	0,98
Difference T1-T0	207,8	782,1	562,9	14,6	0,94

### Difference of intra-examiner measurements (1st-2nd)

The mean ± standard deviation, Dahlberg’s D, coefficient of variation (%), and intraclass correlation coefficient were calculated. The Dahlberg error ranges from approximately 330 to 560 mm3 for the three parameters.

In terms relative to the magnitude of the measured values, the coefficient of variation is good for volumes (2.6% and 3.2%), but debatable for the difference in volumes (14.6%). It should be noted that the difference in volumes accumulates measurement errors from T0 and T1, leading to an increased coefficient of variation.

The intraclass correlation coefficient (ICC) was also calculated as an indicator of reproducibility. The obtained values were quite high (> 0.90), confirming strong agreement and high reproducibility of the measurement method.

## Results

**Fig. 2:**
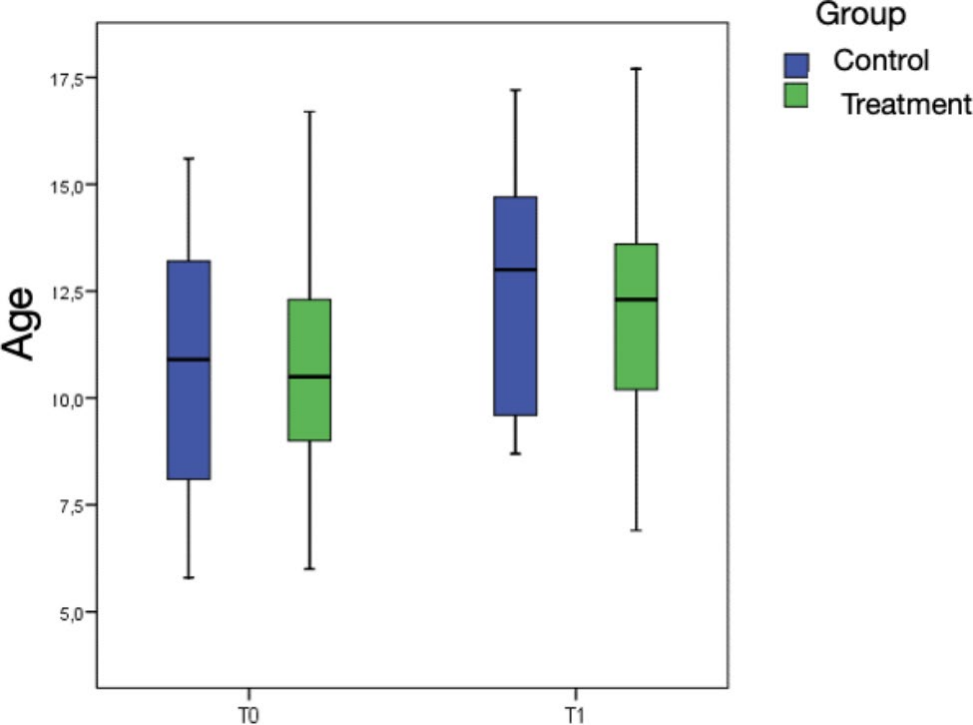
The box in the figure represents the middle 50% of the cases, with the median indicated by a horizontal line dividing it. The upper and lower boundaries of the box represent the 1st and 3rd quartiles, which encompass 25% and 75% of the sample, respectively. The “whiskers” extend to values within an acceptable range, while any data points outside this range are considered outliers (represented by circles) or extremes (represented by asterisks)

Regarding the results of the descriptive variables, it can be stated that there are no significant differences in terms of the starting age (p = 0.958) or final age (p = 0.581). However, there is a tendency (p = 0.087) towards a longer treatment duration in the control group (Fig. 2).

The normality of airway measurements at T0 and T1 in the groups was assessed using the Shapiro-Wilk test, which yielded a confirmatory result (p < 0.05). This confirms that a numerical approach can be used to address the research objectives.

The groups were found to be homogeneous in terms of sex, age at onset, and facial pattern, as indicated in the initial part of the results section. Additionally, the elapsed time between the first and second measurements (follow-up time) in both groups was studied, revealing a slight difference that approached statistical significance (p = 0.087, t-test). This suggests that the controls had a longer follow-up period. This could potentially impact the overestimation of volume values in T1 due to the longer growth time.

Table 3 provides a description of the evolution of airway volume in absolute and relative terms. In the control group, the average volume increased from 10,911.3 ± 1,249.6 mm3 at T0 to 13,168.9 ± 1,789.7 mm3 at T1, indicating an average increase of 2,257.6 mm3 or + 20.9%. In the treated group, the volume increased from 14,126.3 ± 4,399.8 mm3 at T0 to 18,064.1 ± 4,565.9 mm3 at T1, reflecting an average increase of 3,937.8 mm3 or + 31.8%.

**Table 3:** Differences between volume airway in T0 versus T1

	GROUP		
	TOTAL	CONTROL	TREATMENT
**VOLUME T0**			
N	50	13	37
Median	13290,4	10911,3	14126,3
Typical deviation	4078,5	1249,6	4399,8
Minimum	7667,0	9107,8	7667,0
Maximum	24713,8	13192,5	24713,8
Median	12333,6	10765,1	13637,9
**VOLUME T1**			
N	50	13	37
Median	16791,3	13168,9	18064,1
Typical deviation	4561,3	1789,7	4565,9
Minimum	10504,0	10862,9	10504,0
Maximum	26691,0	17945,9	2691,0
Median	15858,4	12989,8	18735,7
**DIFFERENCE VOLUME AIRWAY**			
N	50	13	37
Median	3501,0	2257,6	3937,8
Typical deviation	2503,8	1216,9	2699,1
Minimum	592,0	852,7	592,0
Maximum	10194,0	4753,4	10194,0
Median	2404,0	1905,7	3334,0

In the control group, the average volume increased from 10,911.3 ± 1,249.6 mm3 to 13,168.9 ± 1,789.7 mm3, representing an average increase of 2,257.6 mm3 or + 20.9%. On the other hand, in the treated subjects, the volume increased from 14,126.3 ± 4,399.8 mm3 at T0 to 18,064.1 ± 4,565.9 mm3 at T1. The average increase was estimated at 3,937.8 mm3, equivalent to + 31.8%. These findings suggest that the volume increase is primarily attributable to the therapeutic factor.

The significance level used in the analyses was 5% (α = 0.05). For the described ANOVA model, with a significance level of 5% and considering an effect size of f = 0.35 (medium-large) to be detected, the achieved power is 0.98 for within-subject effects and 0.80 for between-subject effects, as shown in Table 4.

**Table 4:** Changes in volume of the pathway according to Group: F test of the general linear model ANOVA

	p-value
Time	< 0,001***
Group	0,002**
Time x Group	0,036*

*p < 0,05; **p < 0,01; ***p < 0,001

**Fig. 3:**
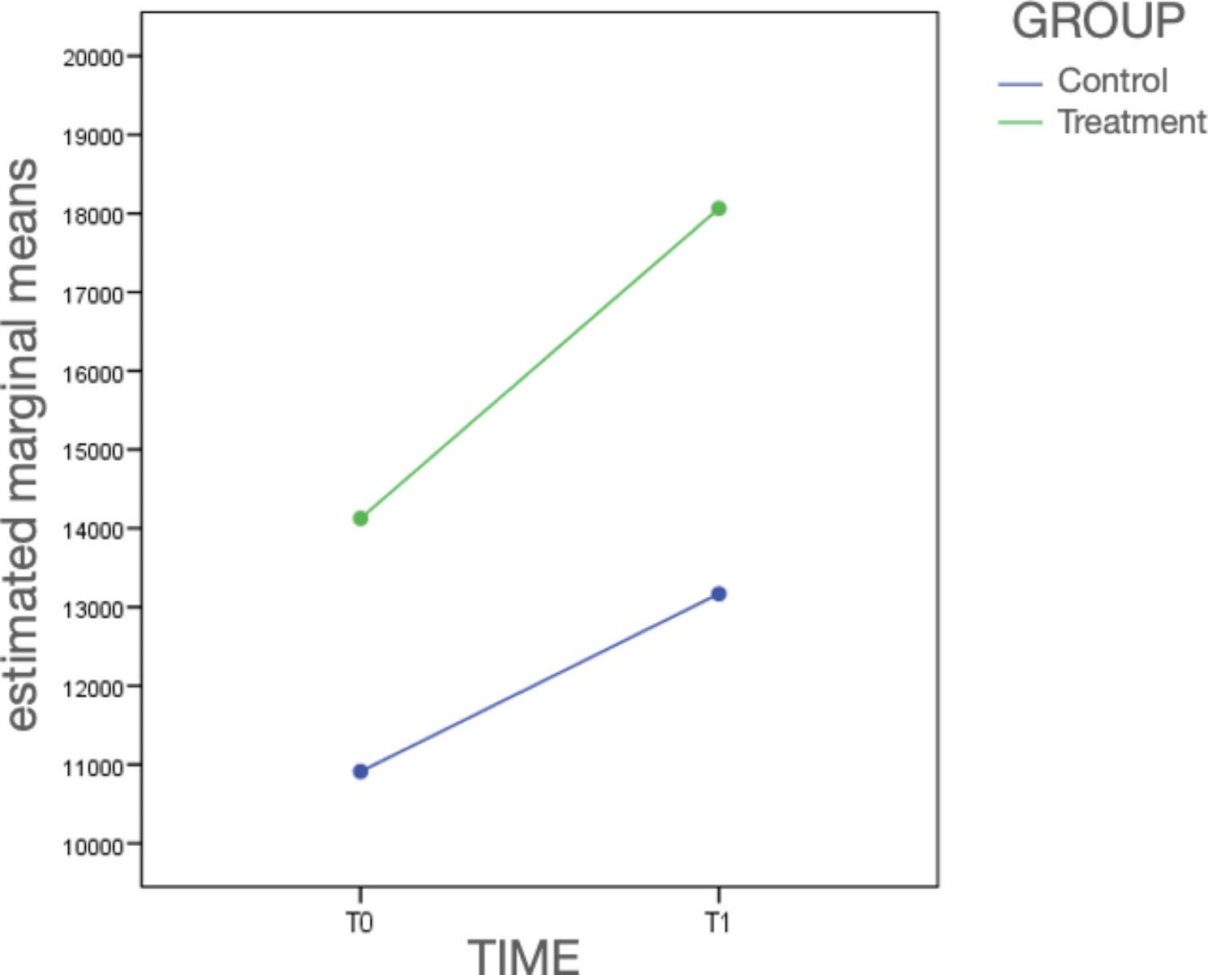
This figure demonstrates that changes in airway volume occur naturally over time, without any specific treatment or intervention

The results provide confirmation that the progression of the airway in the two study groups exhibits a significant difference (p = 0.036), as depicted in Fig. 3.

Furthermore, the observed “time” effect (p < 0.001) indicates that there are overall changes in the airway over time, without distinguishing between the treated and untreated subjects. The interaction effect reinforces that these changes are not identical in both groups.

In summary, the findings confirm that there is a distinct and significant divergence in the evolution of the airway between the two study groups (p = 0.036). Additionally, the “time” effect (p < 0.001) reveals global alterations in the airway over time, while the interaction effect underscores the differential nature of these changes in the treated and untreated subjects.

The Bonferroni tests provide compelling evidence of temporal changes in both the control group (p = 0.001) and the treated group (p < 0.001). These tests also reveal that there were already differences in the airway volume between the control and treated groups at T0 (p = 0.013), which were further emphasized at T1 (p < 0.001). In conclusion, while the control group experienced a significant increase in airway volume, the treated group exhibited a significantly more pronounced advancement.

The sex of the patient did not have any noteworthy impact on the result since there were no significant differences in airway volume between men and women (p = 0.992).

The age of the patients did not show a direct correlation with the final effect. Although there were no differences in terms of the age of onset (p = 0.958) or final age (p = 0.581), there was a tendency towards a longer duration of time in the control group (p = 0.087). The treatment effect (greater volume gain compared to controls) was independent of the age range at which the subjects began treatment (p = 0.757).

No discernible effects were attributed to the individual’s facial profile. The greatest gain in airway volume among treated patients was not dependent on facial pattern (p = 0.427). Additionally, the facial pattern was not associated with larger or smaller volumes of the studied structure (p = 0.871).

It is noteworthy that the control patients were measured with an average time lapse of 1.93 ± 1.02 years, while those who underwent treatment had a time lapse of 1.38 ± 0.64 years. This suggests that the “growth effect” may have been more evident in the control group.

To determine the gain in airway volume specifically attributed to treatment rather than growth, a regression model was developed. This model allowed for quantifying the pure effect of treatment on airway volume. The model explained the T1 volume of the airway in the control group based on T0 volume, age at T0 onset, follow-up time, sex, and facial pattern. By predicting the T1 volume in the treated group using this model, we could assess the volume change solely due to growth. The comparison between the actually measured T1 volume and the growth-predicted volume would determine if there are significant differences, indicating a treatment effect.

The regression model revealed that only the volume at T0 was a significant factor in explaining the final volume (p = 0.015). The predicted T1 volume for the treated group was calculated using the aforementioned regression model. The results are presented in Table 5.

**Table 5:** Changes in volume of the pathway according to the prediction of airway volume that treated patients would have at T0

	GROUP		
	TOTAL	CONTROL	TREATMENT
**VOLUME T0**			
N	50	13	37
Median	13290,4	10911,3	14126,3
Typical deviation	4078,5	1249,6	4399,8
Minimum	7667,0	9107,8	7667,0
Maximum	24713,8	13192,5	24713,8
Median	12333,6	10765,1	13637,9
**VOLUME T1**			
N	50	13	37
Median	16791,3	13168,9	18064,1
Typical deviation	4561,3	1789,7	4565,9
Minimum	10504,0	10862,9	10504,0
Maximum	26691,0	17945,9	2691,0
Median	15858,4	12989,8	18735,7
**VOLUME T1PREDICTED**			
N	37	0	37
Median	16710,7	-	16710,7
Typical deviation	5302,9	-	5302,9
Minimum	8251,1	-	8251,1
Maximum	29840,3	-	29840,3
Median	15141,6	-	15141,6

As shown in Table 4, the treated patients had an average volume of 18,064 mm3 at T1, whereas based on their growth, sex, age, and pattern, they would have been expected to have a volume of 16,711 mm3. This indicates that out of the total volume gain of 3,938 mm3, approximately 2,585 mm3 can be attributed to natural growth factors. The remaining 1,353 mm3 represents the effective gain in volume specifically due to the treatment.

## Discussion

The study sample in this research comprises 50 patients, which is larger compared to other recent studies in the literature, such as those conducted by Alberto Capridoglio in 2014 [20] and Namiko Izuka in 2015 [21]. Specifically, our study includes a treatment group consisting of 37 patients, which contributes to the overall sample size. One unique aspect of our study is the inclusion of a control group, which consists of 13 patients who did not undergo maxillary disjunction treatment for various reasons. This control group provided us with valuable measurements of natural airway development in the absence of treatment, allowing us to determine the specific volume gain attributed solely to the disjunction treatment, which amounted to an average volume increase of 1,353 mm3. Rapid maxillary expansion is a conventional orthopedic treatment commonly performed in orthodontics to address maxillary constriction. It has been investigated as a treatment for sleep disorders, and previous studies have reported an increase in maxillary width and a reduction in nasal resistance and apnea-hypopnea index (AHI) in patients with sleep apnea [9, 11].

In contrast to other published studies, our study exclusively included cases of tooth-supported expanders, which contributed to greater homogeneity within the sample. This focused selection allowed us to investigate the specific effects of tooth-supported expanders on airway expansion.

For example, Kabalan conducted a study in 2015 comparing the differences in airway expansion between bone-supported and tooth-supported expanders, and the findings revealed no significant differences between the two types [15]. Similarly, Baratieri’s study in 2014 exclusively utilized Haas expanders [6], while Zhao’s study focused exclusively on Hyrax expanders [22]. These studies explored the effects of specific types of expanders on airway expansion, whereas our study focused solely on tooth-supported expanders to ensure consistency and provide a clearer understanding of their impact.

As mentioned in the results section of this study, both the treatment group and the control group experienced an increase in airway volume. However, the treatment group exhibited a significantly greater increase compared to the control group, with a difference of 32% between the measurements taken at T0 and T1. These findings align with previous studies that have also reported statistically significant differences in the increases of upper airway volume [1, 6, 15]. The systematic review concluded that there is only a moderate level of evidence supporting the stability of changes induced by rapid maxillary expansion in growing children for a duration of at least 11 months [6]. This suggests that the observed changes in airway volume resulting from the treatment are likely to be maintained over a considerable period of time.

Several studies have reported significant changes in airway volume following maxillary skeletal expansion, leading to improved nasal breathing for a minimum duration of 11 months, during which the changes remained stable [23, 24]. The increase in air volume achieved through maxillary expansion surpassed the baseline measurements observed in the control group. This improvement in airway volume is attributed to the correction of nasopharyngeal obstruction resulting from maxillary disjunction treatment [15, 23].

However, it is important to note that the literature contains studies where an increase in nasal cavity volume was observed without a directly proportional improvement in respiratory function [25, 26]. While an increase in airway volume may suggest a potential benefit for breathing, the relationship between nasal cavity volume and respiratory function is complex and multifactorial. Other factors, such as nasal resistance and airflow dynamics, can influence the overall improvement in respiratory function. Therefore, the impact of changes in nasal cavity volume on actual respiratory outcomes may vary and should be considered in conjunction with additional factors.

In line with the studies mentioned earlier, our study also demonstrated an improvement in airway volume in both the control and treatment groups. However, in the treatment group, we further analyzed the increase in airway volume to differentiate between the portion attributed to natural growth and the portion directly associated with the treatment. This differentiation was accomplished by employing a regression model to estimate the treatment’s specific impact on airway volume.

Unlike the studies mentioned earlier, our study provides a correct estimation of the increase in airway volume. The unique aspect of our study is the comparison between the treatment group and the control group, allowing us to determine the specific impact of the treatment. The observed increase in airway volume was 32% greater than what would have been achieved through natural baseline growth alone. Furthermore, our study implemented a specific protocol for conducting CBCT scans, ensuring that patients were positioned in a natural head posture for accurate measurements. This approach minimizes the potential influence of tongue position on airway volume, as noted by Chang [12].

Fastuca et al. (2015) conducted a cohort study focused on skeletal expansion of the maxilla and its effects on the upper airway volume and sleep-related parameters. They conducted CBCT scans and polysomnography at the beginning and end of the treatment, which involved the removal of the expansion appliance after 12 months. The study demonstrated a significant increase in upper airway volume, along with improvements in oxygen saturation by 5.3% and a reduction in the apnea-hypopnea index by 4.2 fewer events. These findings provide strong support for the effectiveness of maxillary expansion treatment in improving airway function and sleep-related outcomes [1].

## Conclusions

Based on the findings of the study, it can be concluded that palatal disjunction leads to a significant increase in airway volume compared to the control group. The control group showed a mean increase in volume due to growth from 10,911.3 ± 1,249.6 mm3 to 13,168.9 ± 1,789.7 mm3, which corresponds to a mean increase of 2,257.6 mm3 or + 20.9%. In contrast, the treated group experienced a greater increase in volume, going from 14,126.3 ± 4,399.8 mm3 in T0 to 18,064.1 ± 4,565.9 mm3 in T1. The estimated average increase in volume was 3,937.8 mm3, equivalent to + 31.8%. This indicates that the treated subjects achieved a higher volume of 18,064 mm3 in T1, compared to the expected volume of 16,711 mm3 based on their growth, sex, age, and pattern. While these results demonstrate structural changes and an evident increase in airway volume, further investigation is needed to determine the impact of this increase on the actual respiratory capacity and improvement in breathing for the patients.

In summary, the study did not find any significant differences between the treatment and control groups based on factors such as sex, age, facial profile, or pattern. This suggests that the effect of the treatment is not influenced by these variables, and the observed increase in airway volume is independent of the age range at which the subjects began the treatment.

## Data Availability

The datasets generated and/or analyzed during the current study are not publicly available due to confidentiality agreements but are available from the corresponding author on reasonable request.

## References

[1] Fastuca R (2015). Airway compartments volumen and oxygen saturation changes after rapid maxillary expansion: a longitudinal correlation study. Angle Orthod.

[2] Huang YS, Guilleminault C (2017). Pediatric Obstructive Sleep Apnea: where do we stand?. Adv Otorhinolaryngol.

[3] Katyal V, Kennedy D, Martin J, Dreyer C, Sampson W (2013). Paediatric sleep-disordered breathing due to upper airway obstruction in the orthodontic setting: a review. Aust Orthod J.

[4] Garg RK, Afifi AM, Garland CB, Sanchez R, Mount DL (2017). Pediatric Obstructive Sleep Apnea: Consensus, controversy, and Craniofacial Considerations. Plast Reconstr Surg.

[5] Proffit WR (2013). Contemporary orthodontics.

[6] Baratieri Cda L, Alves M, Mattos CT, Lau GW, Nojima LI, de Souza MM (2014). Transverse effects on the nasomaxillary complex one year after rapid maxillary expansion as the only intervention: a controlled study. Dent Press J Orthod.

[7] Galeotti A, Festa P, Viarani V, D’Antò V, Sitzia E, Piga S, Pavone M (2018). Prevalence of malocclusion in children with obstructive sleep apnoea. Orthod Craniofac Res.

[8] Xu Z, Wu Y, Tai J, Feng G, Ge W, Zheng L, Zhou Z, Ni X (2020). Risk factors of obstructive sleep apnea syndrome in children. J Otolaryngol Head Neck Surg.

[9] Ballanti F, Lione R, Baccetti T, Franchi L, Cozza P (2010). Treatment and posttreatment skeletal effects of rapid maxillary expansion investigated with low-dose computed tomography in growing subjects. Am J Orthod Dentofacial Orthop.

[10] Burns DW, Chan VWS, Trivedi A, Englesakis M, Munshey F, Singh M (2022). Ready to scan? A systematic review of point of care ultrasound (PoCUS) for screening of obstructive sleep apnea (OSA) in the pediatric population. J Clin Anesth.

[11] Izuka EN, Feres MF, Pignatari SS (2015). Immediate impact of rapid maxillary expansion on upper airway dimensions and on the quality of life of mouth breathers. Dent Press J Orthod.

[12] Chang Y, Koenig LJ, Pruszynski JE, Bradley TG, Bosio JA, Liu D (2013). Dimensional changes of upper airway after rapid maxillary expansion: a prospective cone-beam computed tomography study. Am J Orthod Dentofacial Orthop.

[13] Camacho M, Chang ET, Song SA, Abdullatif J, Zaghi S, Pirelli P, Certal V, Guilleminault C (2017). Rapid maxillary expansion for pediatric obstructive sleep apnea: a systematic review and meta-analysis. Laryngoscope.

[14] Ronsivalle V, Carli E, Lo Giudice A, Lagravère M, Leonardi R, Venezia P (2022). Nasal septum changes in adolescents treated with tooth-borne and Bone-Borne Rapid Maxillary expansion: a CBCT Retrospective Study using skeletal tortuosity ratio and deviation analysis. Children.

[15] Kabalan O, Gordon J, Heo G, Lagravère MO (2015). Nasal airway changes in bone-borne and tooth-borne rapid maxillary expansion treatments. Int Orthod.

[16] Di Carlo G, Saccucci M, Ierardo G, Luzzi V, Occasi F, Zicari AM, Duse M, Polimeni A. Rapid Maxillary Expansion and Upper Airway morphology: a systematic review on the Role of Cone Beam Computed Tomography. Biomed Res Int. 2017. 5460429. 10.1155/2017/5460429 PMC5534278 28791305

[17] Guijarro-Martínez R, Swennen GR (2011). Cone-beam computerized tomography imaging and analysis of the upper airway: a systematic review of the literature. Int J Oral Maxillofac Surg.

[18] Guinot-Barona C, Soler Segarra I, Arias de Luxán S, Laparra Hernández R, Marqués Martínez L (2022). García Miralles E. A Novel Mathematical Method to diagnose the transverse growth deficit of the Nasomaxillary Complex. Diagnostics (Basel).

[19] Guijarro-Martinez R, Swennen GR (2013). Three-dimensional cone beam computed tomography definition of the anatomical subregions of the upper airway: a validation study. Int J Oral Maxillofac Surg.

[20] Caprioglio A, Meneghel M, Fastuca R, Zecca PA, Nucera R, Nosetti L (2014). Rapid maxillary expansion in growing patients: correspondence between 3-dimensional airway changes and polysomnography. Int J Pediatr Otorhinolaryngol.

[21] Izuka EN, Feres MF, Pignatari SS (2015). Immediate impact of rapid maxillary expansion on upper airway dimensions and on the quality of life of mouth breathers. Dent Press J Orthod.

[22] Zhao Y, Nguyen M, Gohl E, Mah JK, Sameshima G, Enciso R (2010). Oropharyngeal airway changes after rapid palatal expansion evaluated with cone-beam computed tomography. Am J Orthod Dentofacial Orthop.

[23] Ceroni Compadretti G, Tasca I, Alessandri-Bonetti G, Peri S, D’Addario A (2006). Acoustic rhinometric measurements in children undergoing rapid maxillary expansion. Int J Pediatr Otorhinolaryngol.

[24] Cappellette M, Cruz OL, Carlini D, Weckx LL, Pignatari SS (2008). Evaluation of nasal capacity before and after rapid maxillary expansion. Am J Rhinol.

[25] Enoki C, Valera FC, Lessa FC, Elias AM, Matsumoto MA, Anselmo-Lima WT (2006). Effect of rapid maxillary expansion on the dimension of the nasal cavity and on nasal air resistance. Int J Pediatr Otorhinolaryngol.

[26] Gordon JM, Rosenblatt M, Witmans M, Carey JP, Heo G, Major PW, Flores-Mir C (2009). Rapid palatal expansion effects on nasal airway dimensions as measured by acoustic rhinometry. A systematic review. Angle Orthod.

